# Retraction Note: Circular RNA hsa_circ_0001829 promotes gastric cancer progression through miR155-5p/SMAD2 axis

**DOI:** 10.1186/s13046-023-02673-6

**Published:** 2023-04-26

**Authors:** Qiuling Niu, Zhijie Dong, Min Liang, Yuanwei Luo, Hai Lin, Mingzhen Lin, Xiu Zhong, Wenxia Yao, Jinsheng Weng, Xinke Zhou

**Affiliations:** The Fifth Affiliated Hospital of Guangzhou Medical University, Guangzhou Medical University, Guangzhou, China

**Retraction Note:** ***J Exp Clin Cancer Res*** **39, 280 (2020)**

10.1186/s13046-020-01790-w

The Editor-in-Chief has retracted this article. After publication, concerns were raised regarding multiple cases of potential overlap between subpanels in the figures. Specifically:

- Fig. 2E GES-1-T si-NC and Fig. 3D GES-1-T vector images appear to overlap;
- Fig. 2E MKN-28 si-NC, si-circRNA-2#, and Fig. 3D MKN-28 vector images all appear to originate from the same sample, with different rotation in Fig. 3D;
- In Fig. 2F, the MKN-28 24 h images representing si-circRNA-1# and si-circRNA-2# appear to overlap;
- In Fig. 6H, the GES-1-T and MKN-28 vector group images appear to overlap.

Additionally, among the cell lines used in the experiment presented in Fig. 1D, MKN-28, SGC-7901, MGC-803, and BGC-823 cells have been reported to be contaminated.

The authors have stated that the errors in the images occurred during figure preparation, and the results obtained using cell lines contaminated with HeLa cells were only used for validation, thus not affecting the conclusions of the article.

However, due to the high number of concerns with the images, the Editor-in-Chief no longer has confidence in the presented data.

None of the authors have responded to any correspondence from the editor or publisher about this retraction note.

